# MicroRNAs in Diabetic Kidney Disease

**DOI:** 10.1155/2014/593956

**Published:** 2014-01-14

**Authors:** Rong Li, Arthur C. K. Chung, Xueqing Yu, Hui Y. Lan

**Affiliations:** ^1^Li Ka Shing Institute of Health Sciences, The Chinese University of Hong Kong, Shatin, Hong Kong; ^2^Department of Chemical Pathology, The Chinese University of Hong Kong, Hong Kong; ^3^Department of Nephrology, The First People's Hospital of Yunnan Province, Yunnan, China; ^4^Shenzhen Research Institute, The Chinese University of Hong Kong, Shenzhen, China; ^5^Department of Nephrology, The First Affiliated Hospital, Sun Yat-sen University, Guangzhou, China; ^6^Department of Medicine and Therapeutics, The Chinese University of Hong Kong, Hong Kong

## Abstract

Rapid growth of diabetes and diabetic kidney disease exerts a great burden on society. Owing to the lack of effective treatments for diabetic kidney disease, treatment relies on drugs that either reduces its progression or involve renal replacement therapies, such as dialysis and kidney transplantation. It is urgent to search for biomarkers for early diagnosis and effective therapy. The discovery of microRNAs had lead to a new era of post-transcriptional regulators of gene expression. Studies from cells, experimental animal models and patients under diabetic conditions demonstrate that expression patterns of microRNAs are altered during the progression of diabetic kidney disease. Functional studies indicate that the ability of microRNAs to bind 3′ untranslated region of messenger RNA not only shows their capability to regulate expression of target genes, but also their therapeutic potential to diabetic kidney disease. The presence of microRNAs in plasma, serum, and urine has been shown to be possible biomarkers in diabetic kidney disease. Therefore, identification of the pathogenic role of microRNAs possesses an important clinical impact in terms of prevention and treatment of progression in diabetic kidney disease because it allows us to design novel and specific therapies and diagnostic tools for diabetic kidney disease.

## 1. Epidemiology and Diabetes

The prevalence of diabetes is rising worldwide and is expected to reach the devastating number of 439 million by the year 2030 from 285 million in 2010 [1]. This huge elevation is attributed to an escalating tendency towards sedentary lifestyle and westernized choice of diet, leading to obesity. Furthermore, the age of onset for the type 2 diabetic patients is showing a trend to begin in youths [2]. Diabetes is a significant public health concern as its rising incidence has greatly increased the cost of treating both diabetes and its numerous debilitating complications.

## 2. Diabetic Kidney Disease

Diabetic kidney disease is one of the diabetic microvascular complications. Type 1 and type 2 diabetes are distinct in etiology and pathogenesis. In spite of different morphological changes of renal injury in type 1 and type 2 diabetic patients [3], type 1 and type 2 diabetic patients have similar risks of renal injury in diseased kidney [4]. The characteristics of diabetic renal injury includes the effacement of podocyte foot processes, gradual mesangial cell (MC) proliferation and hypertrophy, excessive accumulation of extracellular matrix (ECM) proteins, mesangial expansion, and thickening of the glomerular basement membrane (GBM) [5]. These events eventually lead to nodular glomerulosclerosis (Kimmelstiel-Wilson lesions) [6]. Similar changes occur in the tubulointerstitial, such as tubular hypertrophy, thickening of the tubular basement membrane (TBM), and interstitial fibrosis [6].

The clinical manifestations of diabetic kidney disease are microalbuminuria (30–300 mg/day), followed by macroalbuminuria (>300 mg/day), gradual loss of renal function, and elevation of arterial blood pressure and terminated in renal failure for some patients. Current interventions of diabetic kidney disease including rigorous glycemic control and antihypertensive therapy and angiotensin-converting enzyme inhibitors (ACEIs) and angiotensin II receptor blockers (ARBs) are the first-line drugs. ACEIs and ARBs have been shown to slow down the progression of kidney disease [7–11]. However, there is no effective therapy to halt the progression to the end-stage renal disease (ESRD) after the nephropathy has been established [12].

In addition, diabetic kidney disease is also associated with macrovascular diseases, such as cardiovascular disease. In USA, diabetic kidney disease accounts for almost 50% of all ESRD [6]. Dialysis or kidney transplantation is useful to control uraemia and other symptoms of renal failure in patients with ESRD. But the prognosis for patients with ESRD due to diabetes is not optimistic because less than 50% of patients survive beyond 5 years after diagnosis [13] and the five-year survival rates are similar to those among patients with metastasized gastrointestinal carcinoma [12]. Given the ever-increasing population with diagnosed diabetes and irreversible renal injury after onset of diabetic kidney disease, to develop effective therapy is an urgent need to combat diabetic kidney diseases.

## 3. MicroRNAs

MicroRNAs are short noncoding RNAs 22–25 nucleotides long. As an endogenous production transcript, microRNAs can bind to the 3′ untranslated region (3′UTR) of its target messenger RNA (mRNA) by imperfect complementary manner, leading to posttranscriptional gene silencing. As a result, microRNAs can inhibit gene expression via mRNA degradation, translation inhibition, or transcriptional inhibition [14, 15]. After the discovery of the first microRNA two decades ago, our knowledge of gene regulation and disease mechanisms has been renovated extensively. Nowadays, the critical role of microRNAs has been established in several cellular and biologic processes, such as proliferation, differentiation, and development, and in the regulation of genes relation to immune responses, cancer, and insulin secretion [16–19]. Because microRNAs are vital regulators of gene expression, aberrant of microRNAs are present in human diseases including cancer, hepatitis, and diabetes [17, 20–23]. There are also emerging reports about microRNAs in renal field. Several comprehensive reviews of microRNAs research on kidney development, function, and diseases have been previously published [24–39]. This review will focus on the current research progress of microRNAs in diabetic kidney disease.

## 4. MicroRNAs in the Pathogenesis of Diabetic Kidney Disease

The two cornerstones of progressive diabetic kidney disease are glomerulosclerosis and interstitial fibrosis. Injury of MCs and tubular epithelial cells (TECs) naturally contributed to fibrosis in diabetic kidney disease. Aberrant glucose metabolism results in accumulation of various byproducts such as advanced glycation end products (AGEs), elevation of reactive oxygen species (ROS) and activation of protein kinase C (PKC). All of these events induce TGF-*β* signaling to induce renal fibrosis. Thus, hyperglycemia as a trigger initiates the cascade of renal injury in diabetic kidney disease.

In the biosynthetic process of microRNAs, Dicer is involved in both microRNAs biogenesis and microRNAs-mediated gene silencing [40–43]. As podocytes in the glomerular basement membrane are critical in maintaining the glomerular filtration barrier, podocyte dysfunction will lead to glomerular pathologies as in diabetic nephropathy (DN) or other types of glomerulonephritis [44]. Studies from mice with podocyte-specific deletion of Dicer [45–47] demonstrate that the loss of microRNAs resulted from deletion of Dicer leads to proteinuria, podocyte injury, and renal fibrosis. These results suggest the critical role of microRNAs in the podocytes to maintain the normal renal functions. Today, emerging evidences indicate microRNAs as vital regulators of gene expression during diabetic kidney disease. MicroRNAs involved in diabetic kidney disease were listed in Tables 1 and 2.

### 4.1. miR-192

The first landmark report about the role of microRNA in diabetic kidney disease was performed by Kato et al. [48]. They show that miR-192 levels increase significantly in glomeruli isolated from streptozotocin-injected diabetic mice as well as diabetic db/db mice, in parallel with increased TGF-*β*1 and collagen 1a2 (Col1a2) levels. Upregulation of renal miR-192 during diabetic kidney diseases is also found in db/db mice, type 2 diabetes rat, and whole blood samples of type 2 diabetes patients [49–52]. In addition, TGF-*β*1 induces miR-192 levels in both mouse MCs and isolated glomeruli from both type 1 and type 2 mouse models of diabetes [48, 52]. Other studies also show miR-192 upregulation in MCs and TECs after treatment with high glucose, AGE, and TGF-*β*1 [53–55]. These studies demonstrate that the elevation of renal miR-192 is highly correlated with diabetic condition.

Recent studies demonstrate several possible mechanisms of how miR-192 mediates renal fibrosis. Firstly, miR-192 targets the E-box repressor Smad-1 interacting protein (Sip-1, Sip-1 also called Zeb2) which binds E-box enhancer elements in the *Col1a2 * gene and then promotes collagen deposition in response to TGF-*β*1 [48]. In addition, miR-192 induces expression of miR-216a and miR-217 which target PTEN [56]. Thus, miR-192 can activate Akt kinase to promote fibrosis as Akt activation in mouse MCs induces signatures of diabetic kidney disease, such as ECM gene expression, apoptosis inhibition, and hypertrophy [56]. Furthermore, Kato et al. also report that miR-216a can target Ybx1, an RNA binding protein and a component of P-bodies, and Ybx1 participates in TGF-*β*-induced collagen expression in mouse MCs [57].

The pathological role of miR-192 in diabetic kidney disease is recently confirmed by the miR-192 knockout (KO) mice [58]. Deletion of miR-192 gene in type I diabetic mice reduces albuminuria, proteinuria, renal fibrosis, and hypertrophy when compared to diabetic wild-type mice [58]. Taken together, these studies suggest that miR-192 plays a pathological role in diabetic kidney disease.

The results from animal models exhibit a pro-fibrotic role of miR-192 in diabetic kidney disease [48, 58, 59]. However, the reverse is true in human nephropathy [60, 61]. In patients with established diabetic kidney disease, low expression of miR-192 is correlated with tubulointerstitial fibrosis and low estimated glomerular filtration rate (GFR) [60]. In HK-2 cells, a human proximal TEC line, TGF-*β*1 (10 ng/mL for 96 h) reduces the expression of miR-192 and zinc finger E-box binding homeobox 1 (Zeb1) accompanied by a reduction of E-cadherin and upregulation of zinc finger E-box binding homeobox 2 (Zeb2), PAI-I, and vimentin [60]. Wang et al. also reported that decreased expression of miR-192 and miR-215 by TGF-*β*1 (10 ng/mL for 72 h) in primary rat MCs and TEC, and in the renal cortex of apolipoprotein E knockout mice at 10 weeks of diabetes [61]. These observed differences in murine models may be due to the different models and time points that were used [56, 61]. Reduced miR-192 and miR-215, which target Zeb2, are involved in TGF-*β*/CTGF-mediated changes in E-cadherin expression, demonstrating that miR-192/215 may not affect fibrosis by directly altering the expression of fibrotic markers and ECM proteins [61]. The different findings in expression of miR-192 in human and animal models of diabetic kidney disease reveal the complexity of signaling mechanism during diabetic kidney injury.

### 4.2. miR-21

miR-21 is another profibrotic microRNA because results of *in vitro* studies show that miR-21 expression is upregulated in TECs and MCs after treatment with TGF-*β*1 or under diabetic condition [54, 62–65]. Elevation of miR-21 in renal cortices has been demonstrated in type 1 (OVE26) and type 2 (kk-ay and db/db) diabetic mouse models [63–66], although downregulation of miR-21 expression is reported during early DN in diabetic db/db mice [67]. Recently miR-21 expression has been found to be increased in kidney biopsies from diabetic patients compared to healthy controls [66]. In addition, the pro-fibrotic property of miR-21 is further confirmed by functional analyses as miR-21 positively regulates expression of ECM and *α*-SMA in TECs and MCs after treatment of TGF-*β*1 or under diabetic condition [54, 62–65]. Overexpression of miR-21 in kidney cells also promotes but knockdown of miR-21 reduces renal inflammation under diabetic condition [63].

The exact mechanism of how miR-21 participates in diabetic renal injury may be related to its putative target genes and the activation of TGF-*β* signaling during diabetic condition [68, 69]. MiR-21 may activate the TGF-*β* canonical signaling by suppressing Smad7, an inhibitory Smad [63]. Furthermore, miR-21 may mediate the TGF-*β* noncanonical signaling by targeting Sprouty (SPRY) because SPRY is a potent inhibitor of Ras/MEK/ERK and then suppress TGF-*β*-dependent fibrogenic activities [70]. As phosphatase and tensin homolog (PTEN) is one of potential targets of miR-21 [71, 72], upregulation of Akt pathway may be another mechanism for miR-21 to participate in diabetic kidney injury. Suppression of PTEN by miR-21 is shown to induce phosphatidylinositide 3-kinases (PI3K) and Akt activity, and subsequently induces metalloproteinase-2 (MMP-2) expression [71]. The reciprocal regulation of PTEN levels and AKT1 substrate 1 (PRAS40), a negative regulator of Tor complex 1 (TORC1) activity by miR-21, is shown to mediate critical pathologic features of diabetic kidney disease [64]. During fibrosis, ECM turnover is controlled by both metalloproteinases and tissue inhibitors of metalloproteinases (TIMPs) activities. The findings of TIMP3 as a potential miR-21 target, demonstrate that miR-21 may mediate several pathways to promote renal injury during diabetic kidney diseases [66]. Further studies should be done to clarify how miR-21 regulates inflammation as renal inflammation plays a vital role in diabetic kidney disease [63].

### 4.3. miR-29

Unlike miR-192 and miR-21, miR-29 is an anti-fibrotic microRNA. Expression of miR-29 family (miR-29a, miR-29b, and miR-29c) maintains a high level in normal kidney, lung, and heart [73] and its expression is dramatically reduced in animal models and human samples of fibrotic diseases in heart, lung, and kidney [74–76]. In addition, treatment with TGF-*β*1 or under diabetic condition reduce the expression of the miR-29 family in cultured MCs, TECs, and podocytes and increases expression of ECM proteins [54, 74, 77, 78], indicating that miR-29 may play a protective role during renal injury.

Its protective role against fibrosis has been demonstrated in different disease models. In cell lines from heart, lung, and kidney with TGF-*β* treatment, overexpression of miR-29 suppresses but inhibition of miR-29 promotes expression of fibrotic markers [70, 74, 75, 78–81]. Gene delivery of miR-29b either before or after established obstructive nephropathy successfully blocks progressive renal fibrosis in a mouse model of unilateral ureteral obstruction nephropathy [74], providing a strong support of anti-fibrotic properties of miR-29. Results from heart, lung, and kidneys also demonstrate that the anti-fibrotic effects of miR-29 are mediated through its ability to suppress the ECM-related gene transcription because more than 20 different ECM-related genes have been validated as direct targets of miR-29 by reporter gene assays and some of them are induced by TGF-*β* signaling [75, 80, 82].

The anti-fibrotic properties of miR-29 are also true in diabetic condition that suppression of miR-29a increased the risk of excess collagen deposition [77]. In addition, miR-29a in HK-2 cells negatively regulates collagen IV (Col IV) expression and directly targets the 3′ UTRs of the collagen genes *Col IV a1 * and *Col IV a2 * [77]. On the other hand, miR-29c expression is increased in the diabetic kidneys from type 2 diabetic mouse model. Ectopic expression of miR-29c promotes the progression of diabetic kidney disease via targeting Sprouty homolog-1 and stimulation Rho kinase [49]. This difference may be due to the fact that different kidney disease models may have different expressions of miR-29 family. Thus, we speculate that each miR-29 family member may have distinct biological action on renal tissue remodeling.

### 4.4. miR-200

The well-established function of miR-200 family (miR-200a, miR-200b, miR-200c, miR-429, and miR-141) is to maintain epithelial differentiation and this ability is believed to be the mechanism for miR-200 family to suppress fibrosis [83–85]. These microRNAs are shown to be suppressed in cells that have undergone epithelial to mesenchymal transition (EMT) in response to TGF-*β* [84–87], suggesting that TGF-*β* regulates the expression of these microRNAs to promote EMT. It is generally believed that proximal TECs may undergo EMT to induce renal fibrosis [88]. However, this notion that EMT contributes to renal fibrosis has recently been challenged.

In renal TECs, treatment with TGF-*β*1 and TGF-*β*2 suppresses expression of the miR-200 family in a Smad signaling dependent manner [88, 89]. This reduction is further confirmed in a mouse model of diabetic kidney disease that reduction of renal miR-200a and miR-141 occurs in diabetic kidneys [88], suggesting that miR-200 family plays a protective role in diabetic kidney disease.

However, it is also found that amounts of miR-200b/c are elevated in glomeruli from type 1 (streptozotocin) and type 2 (db/db) diabetic mice and in mouse mesangial cells treated with TGF-*β*1 *in vitro* [52]. Recently, inducing miR-200b and miR-200c and their target FOG2, an inhibitor of phosphatidylinositol 3-kinase activation, is shown to be one of the mechanisms of how TGF-*β* activates Akt in glomerular mesangial cells [90]. The reduction of FOG2 expression is observed in the glomeruli of diabetic mice and TGF-*β*-treated mouse MC. It is unexpected that increase of miR-200b/c levels is detected in diabetic mouse glomeruli and TGF-*β*-treated MC [90]. Transfection with miR-200b/c mimics in MC considerably reduces FOG2 expression and increases cell hypertrophy which is confirmed by FOG2 knockdown in MC. In addition, suppression of FOG2 by miR-200b/c also activates ERKs, which is through PI3K activation [90]. These new findings suggest a new mechanism for TGF-*β*-induced Akt activation through FOG2 suppression by miR-200b/c, which results in glomerular mesangial hypertrophy during DN. However, the differences of miR-200 expression in diabetic kidneys are possibly due to the differences in the origin of cell lines examined, the treatments performed, and the use of different animal models between studies.

### 4.5. MicroRNAs in Glomerular Permeability and Podocytes

Podocytes are epithelial cells of the visceral layer of a renal glomerulus and they play a critical role in maintaining glomerular permselectivity, regulating the synthesis of ECM proteins in glomerular basement membrane (GBM) [5]. It is well accepted that loss of glomerular podocytes, accumulation of extracellular ECM in glomeruli, and hypertrophy and expansion in the glomerular mesangium are key events in the progression of DN [44], resulting in proteinuria and declining function.

Studies from two independent lines of Dicer KO mice generated for podocytes [45, 47] demonstrate that mutant mice developed proteinuria by three weeks after birth and progressed rapidly to end-stage kidney disease. Multiple abnormalities, including foot process effacement, irregular and split areas of the glomerular basement membrane, podocyte apoptosis and depletion, mesangial expansion, capillary dilation, and glomerulosclerosis, were observed in glomeruli of mutant mice [47], demonstrating that proper processing of microRNAs in podocytes is required.

Recently, specific deletion of Dicer in mouse podocyte reveals an enrichment of predicted miR-30 target genes among the upregulated genes [91]. miR-30s are shown to be expressed abundantly in glomerular podocytes in mice and TGF-*β* suppresses expression of *miR-30s * in podocytes [91]. As TGF-*β* expression and TGF-*β* signaling activated during diabetic condition, downregulation of *miR-30s * in podocyte may be responsible of the TGF-*β*-induced podocyte apoptosis.

Urinary podocyte excretion is observed in DN which is related to the reduced adhesive capacity during podocyte damage [92]. Recent studies demonstrate that miR-124 expression is related to adhesive capacity damage of podocyte [93]. During DN, renal miR-124 expression is upregulated in STZ-induced uninephrectomized diabetic rats and suppression of miR-124 ameliorates adhesive capacity of podocyte [93].

Comparative microRNA expression profile arrays identified miR-93 as one of the five microRNAs downregulated in glomeruli from db/db mice as well as in podocytes/microvascular endothelial cells exposed to a high glucose milieu [94]. The decrease in miR-93, targeting VEGF, may also be of relevance for increasing glomerular permeability of DN.

### 4.6. Other MicroRNAs

There are other microRNAs involved in pathogenesis of diabetic kidney disease and we list here the major findings of these microRNAs. Further studies on these microRNAs may shed more light on their roles in diabetic kidney disease.

MiR-25 serves as an endogenous silencer for the NADPH oxidase 4 (Nox4) gene in mesangial cells [95]. Downregulation of miR-25 by high glucose in MCs or in rat diabetic kidney results in the relief of *Nox4 * gene silencing and leads to increased *Nox4 *expression and ROS production [95], suggesting that miR-25 may play a role in protecting the kidney from oxidative stress.

MiR-215 may participate in Wnt/*β*-catenin signaling as miR-215 is dramatically upregulated under diabetic conditions both *in vitro* (MCs) and *in vivo *(db/db mice) [50] and miR-215 mediates TGF-*β*1-induced MC activation and fibronectin expression via a *β*-catenin dependent pathway [50].

MiR-451 protects mesangial hypertrophy as renal miR-451 is downregulated during early diabetic kidney disease in db/db mice by regulating p38 mitogen-activated protein kinases (MAPK) signaling by targeting of Ywhaz (tyrosine 3-monooxygenase/tryptophan 5-monooxygenase activation protein, zeta) [96].

MiR-377 may be involved in fibronectin production and oxidative stress during diabetic kidney disease. Its expression is elevated both in MCs (human and mouse) and in kidney of spontaneous (NOD/Lt) and STZ-induced type 1 diabetes mouse model [53]. MiR-377 targets p21-activated kinase-1 (PAK1) and manganese superoxide dismutase (MnSOD), which indirectly leads to upregulation of fibronectin [53].

MiR-93 is one of signature microRNAs in hyperglycemic conditions [94]. This microRNA is primarily expressed in glomeruli and TECs. However, its expression is inhibited in cultured podocyte cells, renal microvascular endothelial cells, and glomeruli of diabetic mice [94]. In culture, elevated expression of miR-93 reduces vascular endothelial growth factor (VEGF) expression and VEGF is a predicted target of miR-93 [94]. As VEGF targets collagen IV and fibronectin, the repression of miR-93 during diabetic kidney disease may contribute to the production of collagen and fibronectin.

Overall, the aberrant expression of these microRNAs in pathogenesis elicits the critical role of microRNAs in diabetic kidney disease.

## 5. Regulatory Mechanisms of MicroRNA during DN

The exact mechanism of how diabetic condition regulates microRNA expression during kidney diseases is still ongoing. It is believed that TGF-*β* signaling is responsible to promote synthesis of microRNAs during diabetic condition [39]. It has been reported that TGF-*β* signaling enhances the processing of primary transcripts of some microRNAs into its mature form by the Drosha complex [97]. Smad3 physically interacts with Drosha to promote the processing of pri-miR-21 into mature miR-21.

Our laboratory also demonstrates that TGF-*β*/Smad3 signaling mediates the transcription of miR-21, miR-192, miR-433, and the miR-29 family during renal diseases [55, 62, 74, 98, 99]. TGF-*β* inhibits miR-29 expression but stimulates miR-21 and miR-192 expression via the Smad3-dependent mechanism as demonstrated in MCs and TECs knocking down Smad2 or Smad3, or overexpressing Smad7, and in Smad2 or Smad3 KO mouse embryonic fibroblasts (MEF) [54, 55, 62, 74]. In addition, Smad3 physically interacts with Smad binding site (SBE) located in its promoters to regulate the expression of these microRNAs [55, 62, 74, 98]. The ability to regulate TGF-*β*/Smad3-mediated microRNAs via maintaining renal miR-29b but suppressing miR-192 and miR-21 is found to be one of the mechanism of how Smad7, an inhibitory Smad, protects kidneys from fibrosis [54, 55, 100]. This notion is also supported by the results from different mouse models of kidney diseases induced in mice lacking Smad3 or Smad7 or having conditional knockout (KO) for Smad2 or overexpressing renal Smad7 [54, 55, 62, 74, 98].

In addition, a recent study demonstrates that TGF-*β* stimulates a crosstalk circuit between p53 and miR-192 related to the pathogenesis of DN [58]. It is known that expression levels of TGF-*β*1, p53, and miR-192 are increased in expanded glomeruli of diabetic mice [58, 101, 102]. Suppression of miR-192 function *in vivo* inhibits p53 expression in the renal cortex of control and streptozotocin injected diabetic mice [58]. Results from miR-192 KO type I diabetic mice confirm this positive relationship between miR-192 and renal expression of TGF-*β* and p53 [58]. All these results suggest that the TGF-*β*/Smad3 signaling plays an essential role of regulating microRNA expression during DN.

## 6. Therapeutic Potential of MicroRNAs in Diabetic Kidney Disease

In addition to the investigation of the role of microRNAs in DN, the recent focus has shifted to determine the therapeutic potential of the microRNAs in diabetic kidney disease. Restoring expression or inhibition of microRNAs in cells under diabetic condition has shown promising results in suppressing the expression of ECM [63, 65, 96]. Furthermore, *in vivo* delivery of microRNA mimics, inhibitors, or plasmids for overexpressing or knocking down microRNAs in rodent experimental models provides evidences that altering microRNA activity is a possible way to combat diabetic kidney disease. *In vivo* delivery of antagomiR-21 not only ameliorates creatinine clearance ratio and urine albumin creatinine ratio, but also decreases tissue inhibitor of metalloproteinase 1 (TIMP-1), Col IV and fibronectin proteins in kidney of diabetic mice [65]. Gene transfer of *miR-21 * knockdown plasmids into the diabetic kidneys of db/db mice at age 10 weeks significantly ameliorates both microalbuminuria and renal fibrosis and inflammation at age 20 weeks [63]. Knockdown of miR-29c by a specific antisense oligonucleotide restores Sprouty-1 expression and ameliorates albuminuria and mesangial matrix expansion in the type 2 (db/db) diabetic mice [49]. Inhibition of miR-192 with modified antisense oligonucleotides significantly attenuates proteinuria in mice with diabetic kidney disease and suppresses oxidative stress and the renal fibrosis and hypertrophy [59]. Knockdown of miR-215 with antagomiR-215 restores CTNNBIP expression and inhibits Wnt/*β*-catenin signaling and expression of *α*-SMA and fibronectin in the db/db mouse kidney [50]. Overexpression of miR-451 inhibits glomerular MC proliferation* in vivo* [96]. These successful results from rodent diabetic kidney disease models demonstrate two important aspects. Firstly, altering microRNA activity in diabetic kidneys can hold the progression of diabetic kidney diseases which is not shown in current drug treatment. These promising results from experimental models demonstrate the possibility of applying microRNA therapy in the clinical practice. In addition to the conventional plasmid delivery, recent development of chemical modified oligonucleotides, that are stable in the circulation and can freely enter cells to bind to specific microRNA and silence it [30], provides possible and effective delivery methods to ensure the success of microRNA therapy in renal diseases. However, there are still some obstacles for microRNA therapy. Further attention may focus on the risk of off-target effects of microRNAs, specificity of delivery methods, and nonspecific immune response. Therefore, it is still a long way for clinic application with microRNA-based therapy against diabetic kidney disease.

## 7. MicroRNAs as Potential Biomarkers of Diabetic Kidney Disease

Circulating microRNAs in serum, plasma, and urine have been biomarkers of diseases because they can reflect a response to the pathophysiological stresses [103–106]. Investigation of using circulating microRNAs as biomarkers in diabetic kidney disease is ongoing because the delineation of variations of microRNA levels in the body fluids from patients with diabetic kidney disease may provide an understanding of the progression of the disease.

Recent study shows that, when compared urine microRNAs from type 1 patients with persistent and intermittent microalbuminuria, levels of 27 microRNAs are presented at significantly different levels in different stages of untreated nephropathy [107]. These correlations of microRNAs can be mapped to signaling pathways related to renal fibrosis during diabetic kidney disease [107]. A recent present study of assessment about microRNA expression in urinary exosomes from type 1 diabetic patients with and without incipient diabetic nephropathy demonstrates that miR-130a and miR-145 were enriched, while miR-155 and miR-424 reduced in urinary exosomes from patients with microalbuminuria [108]. Interestingly, the increase of urinary exosomal miR-145 levels in an animal model of early experimental diabetic nephropathy is paralleled by miR-145 overexpression within the glomeruli [108]. Treatment with high glucose in cultured MC also upregulates miR-145 levels in both MCs and MC-derived exosomes, suggesting a correlation between circulating microRNA and its renal expression during DN.

MicroRNAs found in circulation or urine appear to be upregulated or downregulated in the progression of diabetic kidney disease. The early detection of their presence in circulation or urine may assist the prediction of the disease course. In order to employ microRNA in the circulation and urine as biomarker for determining the severity of diabetic kidney disease and checking the progress of recovery during treatment, the threshold of detection of microRNAs by various amplification methods should be increased. Establishment of a correlation of patterns of microRNAs that are released into the urine or blood by damaged kidneys and renal specific microRNA expression profiles will be useful in advancing this field by comprehensively determining their relevance in the pathogenesis of diabetic kidney disease.

## 8. Conclusion and Prospective

Recently, microRNAs have emerged as significant post-transcriptional regulators of gene expression in many human diseases [19, 21, 22, 29, 34]. In renal research, more evidence demonstrate that specific microRNAs alter renal physiology by changing expression patterns, mediating actions of TGF-*β* on renal fibrosis, affecting normal functions of MC, TEC, and podocyte, and inducing ECM deposition, podocyte dysfunction, and albuminuria during renal diseases [24, 28, 33, 34]. However, how microRNAs exactly mediate the diabetic renal injury is underexplored. In principle, one microRNA is capable of regulating multiple target genes. The more the reports of how microRNAs participate in renal injury are, the more their targets are identified. For example, more than eight direct targets of miR-21 and more than ten targets of miR-29 are found in renal research. It gives us a confusion which is the exact mechanism of how a specific microRNA is involved in diabetic renal disease. Up to date, target prediction programs can only be a guide line for the potential microRNA targets and the overlap between algorithms is only minimal. The real target needs to be validated experimentally. But the report about the relationship among the target genes of a specific microRNA is scarce and it is still an open field for future microRNA research. Hopefully, the rapid development of high-throughput validation and proteomic analysis can help us to identify real microRNA targets and determine the exact mechanism of microRNAs in renal diseases in near future.

As overexpression or knockdown of individual microRNAs in a specific cell have provided very useful data on their role in renal biology and pathobiology, generation of a single microRNA knockout in a whole-animal or tissue-specific manner should provide valuable information to confirm the critical role of microRNAs in renal physiology or pathology. For example, knockout mice of miR-21 and miR-192 have confirmed their pathological role in kidney diseases. Presence of these animal models should extend our understanding of how microRNAs work* in vivo*.

In addition to development of microRNA therapy and biomarkers, searching the polymorphism of microRNAs may be another important clinic approach to investigate the role of microRNAs in human diseases [109–111].

Finally, microRNAs act as important downstream effectors during diabetic kidney disease. The understanding of the specific role of microRNAs during diabetic kidney disease provides us not only a possible alternative to ameliorate disease progression, but also putative biomarkers for predicting diabetic kidney disease.

## Figures and Tables

**Table 1:** Summary of microRNAs in diabetic kidney disease (upregulation).

microRNA	*In vitro *(cell type)	*In vivo* (animal mode)	Target	References
miR-192	MCs (human, rat, and mouse)	STZ induced DN mice, DN in db/db mice	Sip-1	[48–50, 52–54]
miR-216a miR-217	Primary MCs (mouse)	STZ induced DN mice, DN in db/db mice	PTEN	[56]
miR-216a	Primary MCs (mouse)	STZ induced DN mice, DN in db/db mice	Ybx1	[57]
miR-200b/c	MCs (mouse)	STZ induced DN mice, DN in db/db mice	Zeb1/2	[52, 90]
miR-215	Primary MCs (mouse)	db/db mice	CTNNBIP1	[50]
miR-21	MCs (human and rat) PTEs (mouse)	OVE26 type 1 diabetic mice (12 weeks of age)	PTEN	[64]
	MCs, TECs (rat)	db/db mice (10 or 20 weeks of age)	Smad7	[63]
		kk-ay DN mice (T2DM) (20 weeks of age)	MMP-9	[65]
miR-29c	MCs (mouse)	db/db mice	Sprouty homolog-1	[49]
miR-377	MCs (human and mouse)	spontaneous [(NOD/Lt) mice] and STZ induced DN mice	PAK1 and MnSOD	[53]
miR-192 miR-29c miR-26b miR-200b	Mouse podocytes Mouse kidney Microvascular Endothelial cells			[53]

PTE: proximal tubule epithelial cells; TEC: tubular epithelial cells; MC: mesangial cells; STZ: streptozotocin; DN: diabetic nephropathy.

**Table 2:** Summary of microRNAs in diabetic kidney disease (downregulation).

microRNA	*In vitro *(cell type)	*In vivo* (animal mode)	Target	References
miR-192	PTE human (HK-2 cells)	Patients with established DN	Zeb2	[60, 61]
miR-215	Primary MCs and PTCs (rat)	STZ induced DN in APOE knockout mice (10 weeks of age)	Zeb2	[61]
miR-21	Primary MCs (mouse)	db/db mice (8 weeks of age)	PTEN	[67]
miR-200a	TEC (rat)	STZ induced DN in apolipoprotein E knockout mice	TGF-*β*2	[88]
miR-29a	PTE human (HK-2 cells)		Col IVA1 and Col IVA2	[77]
miR-25	MCs (rat)	STZ induced DN rat	Nox4	[95]
miR-451	Primary MCs (mouse)	Early DN (db/db mice)	Ywhaz	[96]
miR-93	Renal microvascular Endothelial cell Mouse podocytes	db/db mice	VEGF-A	[94]

PTE: proximal tubule epithelial cells; TEC: tubular epithelial cells; MC: mesangial cells; STZ: streptozotocin; DN: diabetic nephropathy.

## References

[1] Shaw JE, Sicree RA, Zimmet PZ (2010). Global estimates of the prevalence of diabetes for 2010 and 2030. *Diabetes Research and Clinical Practice*.

[2] Jones CA, Krolewski AS, Rogus J, Xue JL, Collins A, Warram JH (2005). Epidemic of end-stage renal disease in people with diabetes in the United States population: do we know the cause?. *Kidney International*.

[3] Fioretto P, Caramori ML, Mauer M (2008). The kidney in diabetes: dynamic pathways of injury and repair. The Camillo Golgi Lecture 2007. *Diabetologia*.

[4] Hasslacher C, Ritz E, Wahl P, Michael C (1989). Similar risks of nephropathy in patients with type I or type II diabetes mellitus. *Nephrology Dialysis Transplantation*.

[5] Miner JH (1999). Renal basement membrane components. *Kidney International*.

[6] Kanwar YS, Sun L, Xie P, Liu F-Y, Chen S (2011). A glimpse of various pathogenetic mechanisms of diabetic nephropathy. *Annual Review of Pathology*.

[7] Lewis EJ, Hunsicker LG, Bain RP, Rohde RD (1993). The effect of angiotensin-converting-enzyme inhibition on diabetic nephropathy. *The New England Journal of Medicine*.

[8] Brenner BM, Cooper ME, De Zeeuw D (2001). Effects of losartan on renal and cardiovascular outcomes in patients with type 2 diabetes and nephropathy. *The New England Journal of Medicine*.

[9] Lewis EJ, Hunsicker LG, Clarke WR (2001). Renoprotective effect of the angiotensin-receptor antagonist irbesartan in patients with nephropathy due to type 2 diabetes. *The New England Journal of Medicine*.

[10] The Diabetes Control Retinopathy and nephropathy in patients with type 1 diabetes four years after a Trial of Intensive Therapy. *The New England Journal of Medicine*.

[11] Ziyadeh FN, Sharma K (2003). Overview: combating diabetic nephropathy. *Journal of the American Society of Nephrology*.

[12] Ritz E, Orth SR (1999). Nephropathy in patients with type 2 diabetes mellitus. *The New England Journal of Medicine*.

[13] Blumenthal SS (2011). Evolution of treatment for diabetic nephropathy: historical progression from RAAS inhibition and onward. *Postgraduate Medicine*.

[14] Lee RC, Feinbaum RL, Ambros V (1993). The *C. elegans* heterochronic gene lin-4 encodes small RNAs with antisense complementarity to lin-14. *Cell*.

[15] Wightman B, Ha I, Ruvkun G (1993). Posttranscriptional regulation of the heterochronic gene lin-14 by lin-4 mediates temporal pattern formation in *C. elegans*. *Cell*.

[16] Taganov KD, Boldin MP, Chang K-J, Baltimore D (2006). NF-*κ*B-dependent induction of microRNA miR-146, an inhibitor targeted to signaling proteins of innate immune responses. *Proceedings of the National Academy of Sciences of the United States of America*.

[17] Esquela-Kerscher A, Slack FJ (2006). Oncomirs—microRNAs with a role in cancer. *Nature Reviews Cancer*.

[18] Poy MN, Eliasson L, Krutzfeldt J (2004). A pancreatic islet-specific microRNA regulates insulin secretion. *Nature*.

[19] Bushati N, Cohen SM (2007). MicroRNA functions. *Annual Review of Cell and Developmental Biology*.

[20] Janssen HLA, Reesink HW, Lawitz EJ (2013). Treatment of HCV infection by targeting microRNA. *The New England Journal of Medicine*.

[21] Chang T-C, Mendell JT (2007). MicroRNAs in vertebrate physiology and human disease. *Annual Review of Genomics and Human Genetics*.

[22] Chen C-Z (2005). MicroRNAs as oncogenes and tumor suppressors. *The New England Journal of Medicine*.

[23] Hennessy E, O'Driscoll L (2008). Molecular medicine of microRNAs: structure, function and implications for diabetes. *Expert Reviews in Molecular Medicine*.

[24] Saal S, Harvey SJ (2009). MicroRNAs and the kidney: coming of age. *Current Opinion in Nephrology and Hypertension*.

[25] Kato M, Natarajan R (2009). MicroRNa cascade in diabetic kidney disease: big impact initiated by a small RNA. *Cell Cycle*.

[26] Kato M, Arce L, Natarajan R (2009). MicroRNAs and their role in progressive kidney diseases. *Clinical Journal of the American Society of Nephrology*.

[27] Kaucsár T, Rácz Z, Hamar P (2010). Post-transcriptional gene-expression regulation by micro RNA (miRNA) network in renal disease. *Advanced Drug Delivery Reviews*.

[28] Li JYZ, Yong TY, Michael MZ, Gleadle JM (2010). Review: the role of microRNAs in kidney disease. *Nephrology*.

[29] Akkina S, Becker BN (2011). MicroRNAs in kidney function and disease. *Translational Research*.

[30] Lorenzen JM, Haller H, Thum T (2011). MicroRNAs as mediators and therapeutic targets in chronic kidney disease. *Nature Reviews Nephrology*.

[31] Bhatt K, Mi Q-S, Dong Z (2011). MicroRNAs in kidneys: biogenesis, regulation, and pathophysiological roles. *The American Journal of Physiology: Renal Physiology*.

[32] Ho J, Kreidberg JA (2012). The long and short of microRNAs in the kidney. *Journal of the American Society of Nephrology*.

[33] Kantharidis P, Wang B, Carew RM, Lan HY (2011). Diabetes complications: the microRNA perspective. *Diabetes*.

[34] Kato M, Park JT, Natarajan R (2012). MicroRNAs and the glomerulus. *Experimental Cell Research*.

[35] Chandrasekaran K, Karolina DS, Sepramaniam S (2012). Role of microRNAs in kidney homeostasis and disease. *Kidney International*.

[36] Pandey AK, Agarwal P, Kaur K, Datta M (2009). MicroRNAs in diabetes: tiny players in big disease. *Cellular Physiology and Biochemistry*.

[37] Ma L, Qu L (2013). The function of microRNAs in renal development and pathophysiology. *Journal of Genetics and Genomics*.

[38] Ho J, Kreidberg JA (2013). MicroRNAs in renal development. *Pediatric Nephrology*.

[39] Chung AC, Yu X, Lan HY (2013). MicroRNA and nephropathy: emerging concepts. *International Journal of Nephrology and Renovascular Disease*.

[40] Chendrimada TP, Gregory RI, Kumaraswamy E (2005). TRBP recruits the Dicer complex to Ago2 for microRNA processing and gene silencing. *Nature*.

[41] Gregory RI, Chendrimada TP, Cooch N, Shiekhattar R (2005). Human RISC couples microRNA biogenesis and posttranscriptional gene silencing. *Cell*.

[42] MacRae IJ, Ma E, Zhou M, Robinson CV, Doudna JA (2008). In vitro reconstitution of the human RISC-loading complex. *Proceedings of the National Academy of Sciences of the United States of America*.

[43] Robb GB, Rana TM (2007). RNA helicase A interacts with RISC in human cells and functions in RISC loading. *Molecular Cell*.

[44] Vallon V, Komers R (2011). Pathophysiology of the diabetic kidney. *Comprehensive Physiology*.

[45] Harvey SJ, Jarad G, Cunningham J (2008). Podocyte-specific deletion of dicer alters cytoskeletal dynamics and causes glomerular disease. *Journal of the American Society of Nephrology*.

[46] Ho J, Kar HN, Rosen S, Dostal A, Gregory RI, Kreidberg JA (2008). Podocyte-specific loss of functional microRNAs leads to rapid glomerular and tubular injury. *Journal of the American Society of Nephrology*.

[47] Shi S, Yu L, Chiu C (2008). Podocyte-selective deletion of dicer induces proteinuria and glomerulosclerosis. *Journal of the American Society of Nephrology*.

[48] Kato M, Zhang J, Wang M (2007). MicroRNA-192 in diabetic kidney glomeruli and its function in TGF-*β*-induced collagen expression via inhibition of E-box repressors. *Proceedings of the National Academy of Sciences of the United States of America*.

[49] Long J, Wang Y, Wang W, Chang BHJ, Danesh FR (2011). MicroRNA-29c is a signature MicroRNA under high glucose conditions that targets sprouty homolog 1, and its in vivo knockdown prevents progression of diabetic nephropathy. *The Journal of Biological Chemistry*.

[50] Mu J, Pang Q, Guo Y-H (2013). Functional implications of MicroRNA-215 in TGF-*β*1-induced phenotypic transition of mesangial cells by targeting CTNNBIP1. *PLoS ONE*.

[51] Karolina DS, Armugam A, Tavintharan S (2011). MicroRNA 144 impairs insulin signaling by inhibiting the expression of insulin receptor substrate 1 in type 2 diabetes mellitus. *PLoS ONE*.

[52] Kato M, Arce L, Wang M, Putta S, Lanting L, Natarajan R (2011). A microRNA circuit mediates transforming growth factor-*Β*1 autoregulation in renal glomerular mesangial cells. *Kidney International*.

[53] Wang Q, Wang Y, Minto AW (2008). MicroRNA-377 is up-regulated and can lead to increased fibronectin production in diabetic nephropathy. *The FASEB Journal*.

[54] Chung AC, Dong Y, Yang W, Zhong X, Li R, Lan HY (2013). Smad7 suppresses renal fibrosis via altering expression of TGF-beta/Smad3-regulated microRNAs. *Molecular Therapy*.

[55] Chung ACK, Huang XR, Meng X, Lan HY (2010). miR-192 mediates TGF-*β*/Smad3-driven renal fibrosis. *Journal of the American Society of Nephrology*.

[56] Kato M, Putta S, Wang M (2009). TGF-*β* activates Akt kinase through a microRNA-dependent amplifying circuit targeting PTEN. *Nature Cell Biology*.

[57] Kato M, Wang L, Putta S (2010). Post-transcriptional up-regulation of Tsc-22 by Ybx1, a target of miR-216a, mediates TGF-*β*-induced collagen expression in kidney cells. *The Journal of Biological Chemistry*.

[58] Deshpande SD, Putta S, Wang M (2013). Transforming growth factor-beta induced cross talk between p53 and a microRNA in the pathogenesis of diabetic nephropathy. *Diabetes*.

[59] Putta S, Lanting L, Sun G, Lawson G, Kato M, Natarajan R (2012). Inhibiting microRNA-192 ameliorates renal fibrosis in diabetic nephropathy. *Journal of the American Society of Nephrology*.

[60] Krupa A, Jenkins R, DongLuo D, Lewis A, Phillips A, Fraser D (2010). Loss of microRNA-192 promotes fibrogenesis in diabetic nephropathy. *Journal of the American Society of Nephrology*.

[61] Wang B, Herman-Edelstein M, Koh P (2010). E-cadherin expression is regulated by miR-192/215 by a mechanism that is independent of the profibrotic effects of transforming growth factor-*β*. *Diabetes*.

[62] Zhong X, Chung ACK, Chen H-Y, Meng X-M, Lan HY (2011). Smad3-mediated upregulation of miR-21 promotes renal fibrosis. *Journal of the American Society of Nephrology*.

[63] Zhong X, Chung A, Chen H (2013). miR-21 is a key therapeutic target for renal injury in a mouse model of type 2 diabetes. *Diabetologia*.

[64] Dey N, Das F, Mariappan MM (2011). MicroRNA-21 orchestrates high glucose-induced signals to TOR complex 1, resulting in renal cell pathology in diabetes. *The Journal of Biological Chemistry*.

[65] Wang J, Gao Y, Ma M (2013). Effect of miR-21 on renal fibrosis by regulating MMP-9 and TIMP1 in kk-ay diabetic nephropathy mice. *Cell Biochemistry and Biophysics*.

[66] Fiorentino L, Cavalera M, Mavilio M (2013). Regulation of TIMP3 in diabetic nephropathy: a role for microRNAs. *Acta Diabetologica*.

[67] Zhang Z, Peng H, Chen J (2009). MicroRNA-21 protects from mesangial cell proliferation induced by diabetic nephropathy in db/db mice. *FEBS Letters*.

[68] Meng XM, Chung AC, Lan HY (2013). Role of the TGF-beta/BMP-7/Smad pathways in renal diseases. *Clinical Science*.

[69] Lan HY, Chung AC (2012). TGF-beta/Smad signaling in kidney disease. *Seminars in Nephrology*.

[70] Ding Q, Gladson CL, Wu H, Hayasaka H, Olman MA (2008). Focal adhesion kinase (FAK)-related non-kinase inhibits myofibroblast differentiation through differential MAPK activation in a FAK-dependent manner. *The Journal of Biological Chemistry*.

[71] Roy S, Khanna S, Hussain S-RA (2009). MicroRNA expression in response to murine myocardial infarction: miR-21 regulates fibroblast metalloprotease-2 via phosphatase and tensin homologue. *Cardiovascular Research*.

[72] Thum T, Gross C, Fiedler J (2008). MicroRNA-21 contributes to myocardial disease by stimulating MAP kinase signalling in fibroblasts. *Nature*.

[73] Kriegel AJ, Liu Y, Fang Y, Ding X, Liang M (2012). The miR-29 family: genomics, cell biology, and relevance to renal and cardiovascular injury. *Physiological Genomics*.

[74] Qin W, Chung ACK, Huang XR (2011). TGF-*β*/Smad3 signaling promotes renal fibrosis by inhibiting miR-29. *Journal of the American Society of Nephrology*.

[75] Van Rooij E, Sutherland LB, Thatcher JE (2008). Dysregulation of microRNAs after myocardial infarction reveals a role of miR-29 in cardiac fibrosis. *Proceedings of the National Academy of Sciences of the United States of America*.

[76] Xiao J, Meng X-M, Huang XR (2012). miR-29 inhibits bleomycin-induced pulmonary fibrosis in mice. *Molecular Therapy*.

[77] Du B, Ma L-M, Huang M-B (2010). High glucose down-regulates miR-29a to increase collagen IV production in HK-2 cells. *FEBS Letters*.

[78] Wang B, Komers R, Carew R (2012). Suppression of microRNA-29 expression by TGF-*β*1 promotes collagen expression and renal fibrosis. *Journal of the American Society of Nephrology*.

[79] Du T, Zamore PD (2005). microPrimer: the biogenesis and function of microRNA. *Development*.

[80] Liu Y, Taylor NE, Lu L (2010). Renal medullary microRNAs in dahl salt-sensitive rats: MiR-29b regulates several collagens and related genes. *Hypertension*.

[81] Ye Y, Hu Z, Lin Y, Zhang C, Perez-Polo JR (2010). Downregulation of microRNA-29 by antisense inhibitors and a PPAR-*γ*agonist protects against myocardial ischaemia-reperfusion injury. *Cardiovascular Research*.

[82] Cushing L, Kuang PP, Qian J (2011). miR-29 is a major regulator of genes associated with pulmonary fibrosis. *American Journal of Respiratory Cell and Molecular Biology*.

[83] Howe EN, Cochrane DR, Richer JK (2012). The miR-200 and miR-221/222 microRNA families: opposing effects on epithelial identity. *Journal of Mammary Gland Biology and Neoplasia*.

[84] Gregory PA, Bert AG, Paterson EL (2008). The miR-200 family and miR-205 regulate epithelial to mesenchymal transition by targeting ZEB1 and SIP1. *Nature Cell Biology*.

[85] Korpal M, Lee ES, Hu G, Kang Y (2008). The miR-200 family inhibits epithelial-mesenchymal transition and cancer cell migration by direct targeting of E-cadherin transcriptional repressors ZEB1 and ZEB2. *The Journal of Biological Chemistry*.

[86] Park S-M, Gaur AB, Lengyel E, Peter ME (2008). The miR-200 family determines the epithelial phenotype of cancer cells by targeting the E-cadherin repressors ZEB1 and ZEB2. *Genes and Development*.

[87] Burk U, Schubert J, Wellner U (2008). A reciprocal repression between ZEB1 and members of the miR-200 family promotes EMT and invasion in cancer cells. *EMBO Reports*.

[88] Wang B, Koh P, Winbanks C (2011). miR-200a prevents renal fibrogenesis through repression of TGF-*β*2 expression. *Diabetes*.

[89] Xiong M, Jiang L, Zhou Y (2012). The miR-200 family regulates TGF-*β*1-induced renal tubular epithelial to mesenchymal transition through smad pathway by targeting ZEB1 and ZEB2 expression. *The American Journal of Physiology: Renal Physiology*.

[90] Park JT, Kato M, Yuan H (2013). FOG2 protein down-regulation by transforming growth factor-beta1-induced microRNA-200b/c leads to Akt kinase activation and glomerular mesangial hypertrophy related to diabetic nephropathy. *The Journal of Biological Chemistry*.

[91] Shi S, Yu L, Zhang T (2013). Smad2-dependent downregulation of miR-30 is required for TGF-beta-induced apoptosis in podocytes. *PloS ONE*.

[92] Vogelmann SU, Nelson WJ, Myers BD, Lemley KV (2003). Urinary excretion of viable podocytes in health and renal disease. *The American Journal of Physiology: Renal Physiology*.

[93] Li D, Lu Z, Jia J, Zheng Z, Lin S (2013). miR-124 is related to podocytic adhesive capacity damage in STZ-induced uninephrectomized diabetic rats. *Kidney & Blood Pressure Research*.

[94] Long J, Wang Y, Wang W, Chang BHJ, Danesh FR (2010). Identification of microRNA-93 as a novel regulator of vascular endothelial growth factor in hyperglycemic conditions. *The Journal of Biological Chemistry*.

[95] Fu Y, Zhang Y, Wang Z (2010). Regulation of NADPH oxidase activity is associated with miRNA-25-mediated NOX4 expression in experimental diabetic nephropathy. *American Journal of Nephrology*.

[96] Zhang Z, Luo X, Ding S (2012). MicroRNA-451 regulates p38 MAPK signaling by targeting of Ywhaz and suppresses the mesangial hypertrophy in early diabetic nephropathy. *FEBS Letters*.

[97] Davis BN, Hilyard AC, Lagna G, Hata A (2008). SMAD proteins control DROSHA-mediated microRNA maturation. *Nature*.

[98] Li R, Chung AC, Dong Y, Yang W, Zhong X, Lan HY (2013). The microRNA miR-433 promotes renal fibrosis by amplifying the TGF-beta/Smad3-Azin1 pathway. *Kidney International*.

[99] Zhou Q, Chung AC, Huang XR, Dong Y, Yu X, Lan HY (2013). Identification of novel long noncoding RNAs associated with TGF-beta/Smad3-mediated renal inflammation and fibrosis by RNA sequencing. *The American Journal of Pathology*.

[100] Lan HY, Chung ACK (2011). Transforming growth factor-*β* and smads. *Contributions to Nephrology*.

[101] Kato M, Zhang J, Wang M (2007). MicroRNA-192 in diabetic kidney glomeruli and its function in TGF-*β*-induced collagen expression via inhibition of E-box repressors. *Proceedings of the National Academy of Sciences of the United States of America*.

[102] Putta S, Lanting L, Sun G, Lawson G, Kato M, Natarajan R (2012). Inhibiting microRNA-192 ameliorates renal fibrosis in diabetic nephropathy. *Journal of the American Society of Nephrology*.

[103] Mitchell PS, Parkin RK, Kroh EM (2008). Circulating microRNAs as stable blood-based markers for cancer detection. *Proceedings of the National Academy of Sciences of the United States of America*.

[104] Chen X, Ba Y, Ma L (2008). Characterization of microRNAs in serum: a novel class of biomarkers for diagnosis of cancer and other diseases. *Cell Research*.

[105] Volinia S, Visone R, Galasso M, Rossi E, Croce CM (2010). Identification of microRNA activity by targets’ reverse eXpression. *Bioinformatics*.

[106] Zampetaki A, Willeit P, Drozdov I, Kiechl S, Mayr M (2012). Profiling of circulating microRNAs: from single biomarkers to re-wired networks. *Cardiovascular Research*.

[107] Argyropoulos C, Wang K, McClarty S (2013). Urinary MicroRNA profiling in the nephropathy of type 1 diabetes. *PLoS ONE*.

[108] Barutta F, Tricarico M, Corbelli A (2013). Urinary exosomal microRNAs in incipient diabetic nephropathy. *PloS ONE*.

[109] Chen K, Rajewsky N (2006). Natural selection on human microRNA binding sites inferred from SNP data. *Nature Genetics*.

[110] Saunders MA, Liang H, Li W-H (2007). Human polymorphism at microRNAs and microRNA target sites. *Proceedings of the National Academy of Sciences of the United States of America*.

[111] Zhou J, Peng R, Li T (2013). A potentially functional polymorphism in the regulatory region of let-7a-2 is associated with an increased risk for diabetic nephropathy. *Gene*.

